# Supplementary material for: Repeated noninvasive stimulation of the ventromedial prefrontal cortex reveals cumulative amplification of pleasant compared to unpleasant scene processing: A single subject pilot study
Source: PLoS One. 2020 Jan 21;15(1):e0222057. doi: 10.1371/journal.pone.0222057 (PMC6974138; doi:10.1371/journal.pone.0222057)
Supplement: Attachment — Submitted filename: Response to Reviewers.docx [file pone.0222057.s001.docx]

Dear Dr. Yuan,

Thank you very much for giving us the opportunity to revise our manuscript and to reply to the reviewers’ remarks. In accordance with the suggestions, we have incorporated a number of changes into the manuscript, and believe the final product is improved considerably.

The data presented in the manuscript might be shared upon request. There are no legal or ethical restrictions. However, due to the size of the data set, we refrain from directly uploading it.

Please consult below our point-to-point replies to the reviewers’ comments.

Reviewer #1

However, it seems a bit unclear about the passive viewing procedure. I am a bit confused whether the passive viewing task uses the same pictures in each tDCS sessions, or different pictures in each session? How the valence of pictures balanced? Besides, the study design without a sham tDCS group, how to prevent the placebo effect of the subject, especially under the non-blind condition?

I am a bit confused whether the passive viewing task uses the same pictures in each tDCS sessions

We thank the reviewer for this comment and added more information about the task. The passive viewing task featured the same set of stimuli across all 20 MEG measurements in different randomizations. This information has now been added to the manuscript (line 112-114). The same approach was administered in our previous studies [1–3] as it controls for physiological variance induced by differences of stimulus characteristics. As for possible habituation effects, it is well known that processing of highly emotional stimuli results in increased event-related potentials compared to neutral stimuli that remain stable across several repetitions, e.g. [4,5].

How the valence of pictures balanced?

Both categories (pleasant and unpleasant) consisted of three subcategories (pleasant: 16 x erotica, 8 x romantic couples, 8 x happy children and families; unpleasant: 16 x graphic bodily injuries, 8 x threatening animals, 8 x threatening humans). The aforementioned stimuli have been rated previously by two independent student samples for—amongst others—perceived valence on a visual analog scale. In both samples, pleasant content received significantly higher pleasantness ratings than unpleasant content.

Besides, the study design without a sham tDCS group, how to prevent the placebo effect of the subject, especially under the non-blind condition?

We agree with the reviewer. A proper blinding of participants can be viewed as part of standard study protocols in tDCS research. However, in this pilot study, we intentionally abstained from blinding our participant. Therefore, in this pilot study we cannot eliminate the possibility of any kind of implicit placebo influences. However, the reason why we chose to conduct the study without any blinding and the assumed consequences of this choice are described in the manuscripts introduction (see line 64-70: *„* *With respect to a planned extension of this study to healthy controls, a further goal was to identify possible negative physiological or psychological side effects of repeated stimulation such as headaches, dysphoric- or depression-like symptoms during or after repeated inhibitory, or euphoria or mania-like symptoms during or after repeated excitatory stimulation. Weighing false positive less critical than false negative findings of detrimental side effects, we decided against a blinding of tDCS conditions, i.e. with distinct knowledge of potential side effects we expected a lower detection threshold.”)* and in the limitation section (see line 238-251 *“Due to the intentional non-blinding of MJ to the tDCS conditions, it is possible that focused top-down attention—although unintentional—might have increased valence specific processing as for instance shown for explicit directed attention by Schupp and coworkers for emotional scenes [33] or Schindler and Kissler [34] for emotional words. It is also possible that implicit, unintentional reappraisal processes have influenced scene valence perception. For instance, Li and colleagues [35] presented results of successful upregulation of pleasant stimulus material. With regard to the cumulative effects, a study of Denny and Ochsner [36] pointed to possible additive effects of downregulation of negative emotions over the course of four sessions. However, in that study, two reappraisal methods were compared and only one showed cumulative effects that were not attributable solely to habituation. It therefore might require a clear regulation strategy in order to cumulatively downregulate emotions over sessions. MJ, in contrast, passively viewed all presented images without explicitly directing attention to one specific category and without applying any specific strategy. Nevertheless, as in our previous studies, follow-up studies should keep the participants blind with regard to underlying hypotheses and stimulation conditions.”*).

Reviewer #2

1) One big problem is the reliability and repeatability of the result. All findings were from a case, lacking of control condition that limits the interpretation and conclusions about the clinical significance of the results. The interpretation of the results is speculative, as mentioned in the limitations, these might be explained by inter-individual differences, and need to be tested in a representative sample.

We thank the reviewer for this comment and agree. The data presented here does only allow for a preliminary interpretation and therefore conclusion. A next step would be to apply this paradigm in a representative sample in a double-blind design to investigate if these effects are (1) independent of any placebo effects and (2) visible across individuals. However, as mentioned in the manuscript, we think that it is worth to share this data with the community, as data availability on tDCS mechanisms like cumulation effects as well as tDCS in context of emotional processing is still low and might therefore help promoting research in this area. Especially the observation that repeated stimulations did not lead to more than mild negative side effects appears important to ethically justify future studies of repeated stimulation in healthy controls. Nevertheless, we want to emphasize that the limitations of this study have been made transparent and the restriction of a single subject study is mentioned in the conclusion as well as in the title.

2) The introduction seems to be too short and too vague. The authors need to provide a more comprehensive theoretical and empirical literature review of the associations between emotional modulation and repeated tDCS to highlight the importance of repeated tDCS in emotional processing research

We are thankful for this annotation and therefore extended the introduction section (line 52-59). Normally, non-invasive brain stimulation techniques like tDCS and TMS in clinical trials are applied across several sessions over a period of one up to several weeks [6,7]. Thus, this information has now been added to increase intelligibility of why it is important to investigate the possible cumulative effects and test for side effects in a repeated stimulation design of this new tDCS protocol.

3) The interval between sessions of inhibitory stimulation and sessions of excitatory stimulation seems too long (one month), why?

We thank the reviewer for this annotation. This interval length was chosen based on tDCS literature that investigated the outcomes of various—much shorter—intervals between consecutive tDCS sessions [8,9]. Due to possible unwanted carryover effects, we aimed to prevent the influence from one stimulation condition on the other. Therefore, we chose an interval that can be considered quite long in relation to other tDCS studies. Of course, similar to many other tDCS studies (1) the chosen interval length has been arbitrary and (2) does not allow for an absolute elimination of such carryover effects. However, it is difficult to ascertain an ideal interval between both conditions in a within-subjects design. Certainly, an interval might as well be too long (due to normal physiological changes over time), as it also might be too short (due to possible carryover effects). However, this would be a question on its own that should be investigated in a separate study.

4) How long did each stimulus present? How long is the interval between stimuli? More details are needed to describe the viewing task in Procedure.

We apologize for our brevity in the Methods-section with regard to the passive viewing task. More information about the stimuli (line 97-102) as well as about the passive viewing task itself (line 104-114) has now been included in the manuscript.

5) Could you give further elaboration of results? It may be more readable and clearer to combine the analysis and result part.

We now subdivided the description of the statistical analysis of MEG data into three subsections by adding the labels Hypothesis 1-3. The same labels are used in the Results-section to increase readability and intelligibility. However, we would like to refrain from combining the results and the methods section directly, as we are afraid that this might decrease readability and intelligibility.

6) In the discussion, author mentioned “While such implicit effects might potentially explain the non-cumulative effects across sessions it is unlikely to explain the cumulation of effects”, can you give a clearer explanation?

We apologize for the confusion caused by this sentence. It has now been removed to increase intelligibility. An explanation for why a cumulative effect as result of implicit, involuntary processing seems rather unlikely is given further down below (line 241-250). As reported by Denny and Ochsner [10], it might be necessary to apply a clear regulation strategy to cumulatively upregulate specific stimuli across consecutive sessions.

7) The authors admit the limitation of the study in that non-blinding design may cause additional influence such as attention. What ways do the authors consider can be used to examine or control this influence? Also, in the further study, single-blind or double-blind design might be more proper.

As it has been described in the manuscript, an important step would be the blinding of all participants to prevent implicit attention or appraisal effects caused by the knowledge about underlying hypotheses. To further subtract mere placebo effects, it is recommendable to investigate a sham condition in addition to active stimulation conditions. For a future study design, we would therefore propose to investigate repetition effects of vmPFC-tDCS with conditions excitatory, inhibitory and sham. However, though this study does not speak against repeated tDCS in healthy controls, as first test we would recommend a single tDCS repetition on two consecutive days and a systematic assessment of side effects and symptoms of stimulations. This information has now been added to the manuscript (line 256-260).

**References**

1.	Junghofer M, Winker C, Rehbein MA, Sabatinelli D. Noninvasive stimulation of the ventromedial prefrontal cortex enhances pleasant scene processing. Cereb Cortex. 2017;27: 3449–3456. doi:10.1093/cercor/bhx073

2.	Winker C, Rehbein MA, Sabatinelli D, Dohn M, Maitzen J, Wolters CH, et al. Noninvasive stimulation of the ventromedial prefrontal cortex modulates emotional face processing. Neuroimage. 2018;175: 388–401. doi:10.1016/j.neuroimage.2018.03.067

3.	Winker C, Rehbein MA, Sabatinelli D, Dohn M, Maitzen J, Roesmann K, et al. Noninvasive stimulation of the ventromedial prefrontal cortex indicates valence ambiguity in sad compared to happy and fearful face processing. Front Behav Neurosci. 2019;13: 1–16. doi:10.3389/fnbeh.2019.00083

4.	Schupp HT, Stockburger J, Codispoti M, Junghofer M, Weike AI, Hamm AO. Stimulus novelty and emotion perception: the near absence of habituation in the visual cortex. Neuroreport. 2006;17: 365–369. doi:10.1097/01.wnr.0000203355.88061.c6

5.	Codispoti M, Ferrari V, Bradley MM. Repetitive picture processing: autonomic and cortical correlates. Brain Res. 2006;1068: 213–220. doi:10.1016/j.brainres.2005.11.009

6.	Lefaucheur J-P, Antal A, Ayache SS, Benninger DH, Brunelin J, Cogiamanian F, et al. Evidence-based guidelines on the therapeutic use of transcranial direct current stimulation (tDCS). Clin Neurophysiol. 2017;128: 56–92. doi:10.1016/j.clinph.2016.10.087

7.	Perera T, George MS, Grammer G, Janicak PG, Pascual-Leone A, Wirecki TS. The clinical TMS society consensus review and treatment recommendations for TMS therapy for major depressive disorder. Brain Stimul. 2016;9: 336–346. doi:10.1016/j.brs.2016.03.010

8.	Monte-Silva K, Kuo M-F, Liebetanz D, Paulus W, Nitsche MA. Shaping the optimal repetition interval for cathodal transcranial direct current stimulation (tDCS). J Neurophysiol. 2010;103: 1735–1740. doi:10.1152/jn.00924.2009

9.	Monte-Silva K, Kuo M-F, Hessenthaler S, Fresnoza S, Liebetanz D, Paulus W, et al. Induction of late LTP-like plasticity in the human motor cortex by repeated non-invasive brain stimulation. Brain Stimul. 2013;6: 424–432. doi:10.1016/j.brs.2012.04.011

10.	Denny BT, Ochsner KN. Behavioral effects of longitudinal training in cognitive reappraisal. Emotion. 2014;14: 425–433. doi:10.1037/a0035276
